# Primary leiomyosarcoma of the thyroid with concurrent papillary thyroid cancer: a rare case report and a review of literature

**DOI:** 10.1186/s13044-023-00157-5

**Published:** 2023-06-05

**Authors:** Mohamed Asiri, Faisal Alsarrani, Abdullah Altasan, Faisal Alqahtani, Lujain Akram Ali, Majed Pharaon, Saad Alshehri, Awad Alshahrani

**Affiliations:** 1 College of Medicine, King Saud Bin Abdulaziz University for Health Sciences, Riyadh, Saudi Arabia; 2 King Abdullah International Medical Research Center, Riyadh, Saudi Arabia; 3 Department of General Surgery, Ministry of National Guard – Health Affairs, Riyadh, Saudi Arabia; 4 College of Medicine, Umm Al-Qura University, Makkah, Saudi Arabia; 5 Department of Pathology and Laboratory Medicine, Ministry of National Guard – Health Affairs, Riyadh, Saudi Arabia; 6 Department of Medicine, Ministry of National Guard – Health Affairs, Riyadh, Saudi Arabia

**Keywords:** Thyroid leiomyosarcoma, Papillary thyroid cancer, Primary thyroid soft tissue tumor, Primary leiomyosarcoma, Thyroid nodules

## Abstract

**Background:**

Leiomyosarcoma (LMS) is a soft tissue malignant tumor that has a predilection to the abdominopelvic and limb smooth muscles. LMS of the thyroid is exceptionally rare. Papillary thyroid cancer (PTC) is the most common thyroid malignancy and originates from the thyroid epithelial layer. To our knowledge, the presence of both tumors in the same patient has not been reported previously.

**Case presentation & literature review:**

A 42-year-old woman presented with a progressively enlarging neck mass for a few months. She underwent left thyroid lobectomy, and the histology showed high-grade primary LMS of the thyroid. She subsequently underwent a complete thyroidectomy, which identified a classical PTC on her right lobe.

Our comprehensive literature review identified 39 published cases of primary LMS of the thyroid. The average tumor size was 5.88 cm and occurred more in women. The most common presentation was neck mass, followed by compressive symptoms. Recurrence and metastasis were uncommon at 15% and 10–25%, respectively.

**Conclusion:**

Thyroid LMS is a rare malignancy with a worse prognosis than PTC. A thorough workup must be done to rule out metastasis before labeling it as primary thyroid cancer.

## Background

Leiomyosarcomas (LMS) are malignant tumors of soft tissues that are mesenchymal in origin [1]. They are commonly found in the trunk, most commonly in the pelvis and gastrointestinal tract, extremities, and head and neck [2]. They account for less than 1% of all adult malignancies [2]. An unusual place to find primary LMS is within endocrine organs such as the thyroid. Thyroid cancer is a common neoplasm with an incidence of 13.5 new cases per 100,000 per year [3]. 80% of thyroid malignancy is classified as papillary thyroid cancer (PTC) [4]. PTCs arise from the epithelial layer of the thyroid and have an excellent 5-year survival prognosis [4]. On the other hand, sarcomas of the thyroid are exceedingly rare, comprising almost 0.014% of primary thyroid cancers [5]. Primary LMS cases of the thyroid have been reported in the literature. It has a poor prognosis, unlike that of PTC and there is little to no consensus on appropriate management and diagnosis. To diagnose such a rare malignancy, there must be an extensive investigation into the histopathology of the tumor with immunohistochemical staining, as well as thorough imaging to look for the possible site of the tumor to rule out metastasis [5]. We present a case of a patient who had both primary LMS in the left lobe, and PTC in the right lobe of her thyroid. To our knowledge, this is the first reported case of both tumors in the same patient in the literature.

## Case presentation

A 42-year-old woman presented to our clinic with a progressively enlarging neck mass for a couple of months. There were no associated compressive symptoms, swallowing issues, or other thyroid disease-related symptoms. There was no history of weight loss or night sweats. A review of her systems was unremarkable. In the past, she had undergone cholecystectomy, sleeve gastrectomy, breast implantations, and benign uterine fibroids excision with continuous monitoring. She had no family history of thyroid disorders or malignancy. On examination, she looked well with no scars or signs of cachexia. She had a rubbery submandibular goiter with no skin changes or palpable lymph nodes. TSH was found to be 1.22 mIU/L (reference range 0.5 to 5.0 mIU/L) and free T4 11.93 pmol/L (reference range 12 to 30 pmol/L). Ultrasound of the neck revealed a Thyroid Nodule Image Reporting and Data Systems (TI-RADS) 5 thyroid nodule with a size of 3.89 × 2.4 × 2.1 cm in the left lobe, requiring a fine needle aspiration (FNA) which was performed 2 days later, and a much smaller nodule with a size of < 1.1 cm. FNA findings revealed an atypia of undetermined significance that classifies it as Bethesda category 3. She was advised to undergo surgery to remove the left lobe. After considering the risks and benefits of the procedure, she underwent left hemithyroidectomy a week later. A conventional surgical approach was utilized with a midline central incision, and the operation took roughly 2 h.

### Specimen description and subsequent surgery

**Fig. 1:**
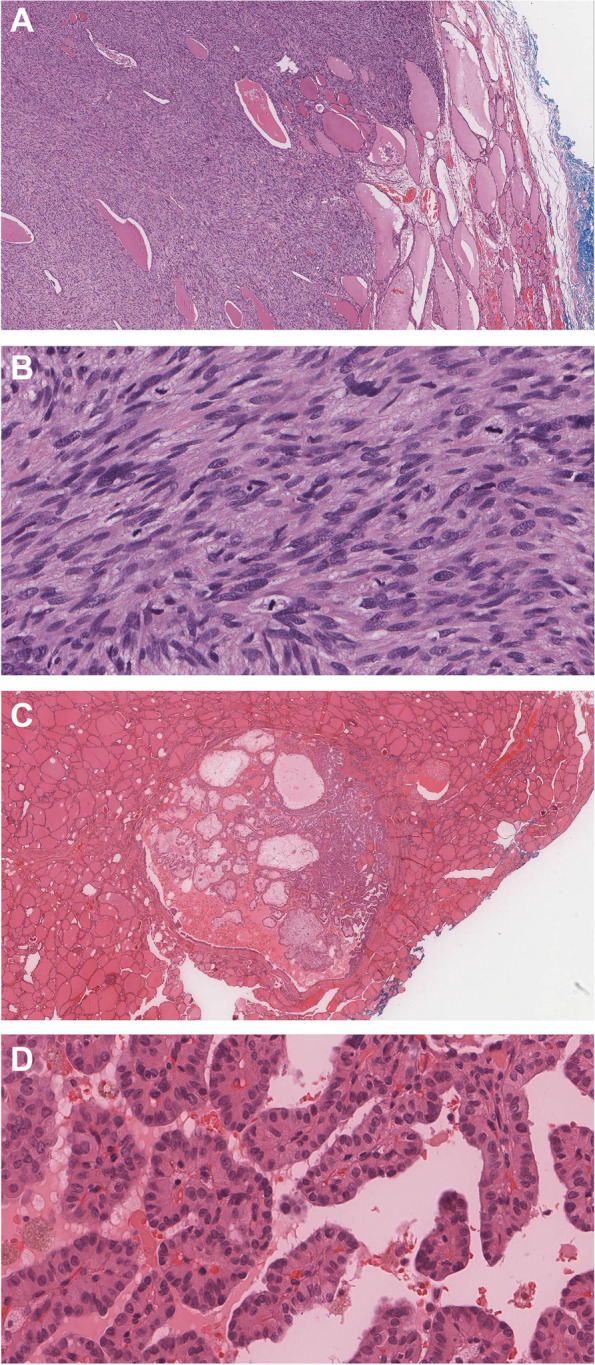
**A**-**B** Leiomyosarcoma in left lobe. **A** Spindle cell tumor abutting and infiltrating thyroid follicles (H&E*, original magnification × 40). **B** Malignant spindle cells with frequent mitotic figures (H&E*, original magnification × 400). **C-D** Papillary thyroid carcinoma (PTC) in right lobe. **C** Small well-circumscribed PTC (H&E*, original magnification × 40). **D** Papillary architecture with nuclear features of PTC (H&E*, original magnification × 400) *Hematoxylin and Eosin

**Fig. 2:**
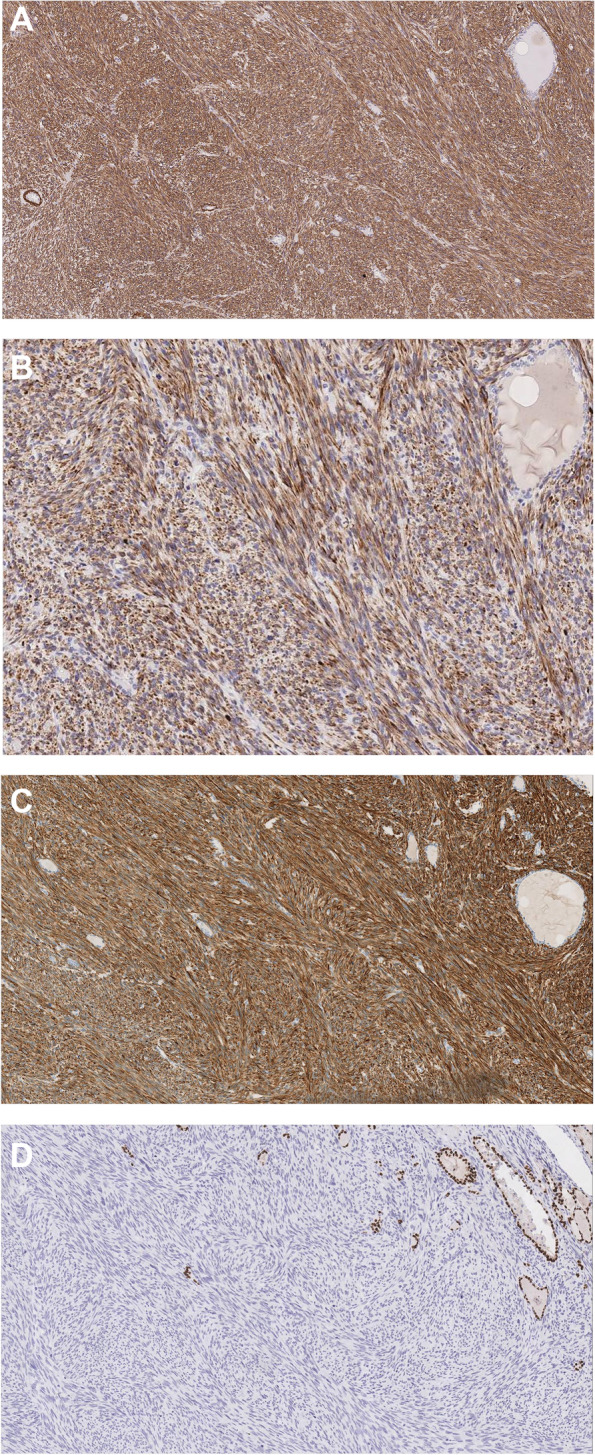
Immunohistochemical staining of the leiomyosarcoma (original magnification × 100). Positive staining for SMA (**A**), Desmin (**B**), and Caldesmon (**C**). TTF-1 is negative in tumor cells while highlighting the nuclei of entrapped follicular cells (**D**)

Gross examination revealed a white well-defined solid mass occupying the majority of the lobe and measuring 4.5 cm in the greatest dimension. The extrathyroidal extension of the tumor into fibro-adipose tissue only was noted. No muscle involvement was seen. Microscopically, the mass consisted of atypical spindle cells arranged in a fascicular growth pattern. The spindle cells showed eosinophilic fibrillary cytoplasm and focal granularity while the nuclei were cigar-shaped with blunt ends and showed variable degrees of atypia such as irregularity, hyperchromasia, and enlargement. Mitotic figures were more than 20 mitoses per 10 high power fields (> 20/10 HPF) (Fig. 1, A-B). An extensive panel of immunohistochemical stains was performed to determine the type of these spindle cells. Smooth Muscle Actin (SMA), caldesmon, and desmin were strongly and diffusely positive, indicating a smooth muscle origin (Fig. 2, A-C). The epithelial markers pan-cytokeratin, CAM5.2, CK7, CK19, and CK5/6 were negative. While the differential diagnosis of medullary thyroid carcinoma was considered due to the presence of spindle cells, the calcitonin stain was negative. Also, the thyroid-specific markers such as Thyroid Transcription Factor (TTF-1) (Fig. 2, D) and PAX-8 were non-reactive. Other markers such as S-100, ERG, myogenin, and STAT-6 were also negative, ruling out the possibility of neural and vascular markers, rhabdomyosarcoma, and solitary fibrous tumor. The overall findings were consistent with a high-grade leiomyosarcoma. As she had small nodules in the remaining lobe of the thyroid seen on ultrasound, she requested and underwent a completion thyroidectomy. The histology showed an incidental microscopic focus of classic papillary thyroid carcinoma (PTC) measuring 0.5 cm in the greatest dimension. The tumor cells were arranged in a papillary architecture and displayed crowding, elongation, overlapping, vesicular chromatin, nuclear grooves, and rare intranuclear pseudo-inclusions (Fig. 1, C-D). The patient was advised oncology and gynecology review. Her gynecology review ruled out malignant uterine, cervical, and ovarian disease, suggesting further that the tumor was primary. She had longstanding small uterine fibroids. There was no history of vaginal bleeding, menorrhagia, or dysmenorrhea. The Pap smear was negative. Whole-body CT was followed up and showed no further lesions other than clinically insignificant previously known pulmonary nodules. The patient has been feeling well, and remains under our regular reviews a year later.

## Discussions and conclusions

Leiomyosarcoma (LMS) of the thyroid is an extremely rare malignancy, and to have a concurrent papillary thyroid cancer adjacent to it makes it even more extraordinary. We found 39 [5–39] reported cases in the literature, with our current case being the 40^th^, and have analyzed common findings and outcomes within each paper (Table 1). The mean tumor size from available data was 5.88 cm., with the largest tumor measuring 13.5 cm [6]. It is more common in females, with 64.1% out of 39 cases that stated gender were women. Two pediatric cases were reported by Ramakrishnan et al. and Tulbah et al., with the latter demonstrating a rare congenital immunodeficiency with Epstein-Barr virus-induced LMS in multiple organs including the liver, thyroid, and right lung [7, 8]. Only one previous case reported coexisting benign uterine fibroids like in our patient [9]. Four cases illustrated previous history of malignancies [6, 10–12]. Piana et al. [12] reported a case who had a previous uterine myxoid LMS and presented 4 years later with thyroid LMS. They argued that the patient’s history strongly indicates a metastasis, however, it was a solitary lesion that had different characteristics from the uterine tumor. They also proposed that there is a chance the patient had two different kinds of LMS originating within two different organs, purely by coincidence.

**Table 1:** Overview of Thyroid Leimyosarcoma Cases in the Literature

Author Ref	Date	Gender	Age (years)	Presenting Complaint	Tumor Size in cm	Outcome	Treatment (Surgery, Radiotherapy, Chemotherapy)	Previous malignancy?
[5] Thompson	1997	F	64	﻿Multiple Nodules	7.5	﻿DWD after 5 months	Surgery	-
[5] Thompson	1997	M	45	Mass, Weight Loss	9	﻿Alive, NLM	Surgery, Chemotherapy	-
[5] Thompson	1997	M	68	﻿Mass, Compression Symptoms	1.9	﻿DWD after 18 months, NLM	?	-
[5] Thompson	1997	M	83	﻿Mass, Compression Symptoms	5.5	﻿DWD after 3 months, NLM	Surgery	-
[6] Zou	2016	M	83	Mass, Compression Symptoms	13.5	DWD after 5 months, Recurrence	Surgery, Chemotherapy	Thyroid Carcinosarcoma, Prostate Cancer
[7] Ramakrishnan	2002	F	3	Mass	3.5	?	Surgery	-
[8] Tulbah	1999	?	PED	??	?	NLM	?	EBV LMS
[9] Kawaguchi	1990	F	72	Mass, Compression Symptoms	?	DWD	None	Uterine Fibroids
[10] Just	2008	F	83	Mass, Pain in the Arm	6.7	DWD after 2 months, LA	Palliative	Colorectal Cancer, Breast Cancer
[11] Vujosevic	2019	F	60	Mass, Compression Symptoms	2.5	Alive, LA, LM, NLM	Surgery, Radiotherapy, Chemotherapy	Uterine Endometrial Adenocarcinoma
[12] Piana	2011	F	59	Mass	?	Alive	Surgery	Uterine Myxoid Leiomyosarcoma
[13] Mansouri	2008	F	63	Mass, Weight loss, Compression Symptoms	7	DWD after 5 months, NLM	Surgery	-
[14] Sahin	2016	M	39	Weight Loss, Compression Symptoms	2.5	DWD after 3 months, NLM	Radiotherapy as Palliative	-
[14] Sahin	2016	F	72	Mass, Compression Symptoms	?	DWD after 1.5 months, LA, LM, NLM	Surgery	-
[15] Wang	2008	F	65	Mass, Weight Loss, Cough	7.5	DWD after 4 months, LA, LM	Surgery, Chemotherapy	-
[16] Adachi	1969	F	74	Mass, Compression Symptoms, Anorexia, Weight Loss	12	DWD after 1 month, LA, LM, NLM	Chemotherapy	-
[17] Mouaqit	2013	M	65	Left arm pain	9	Alive	Surgery	-
[18] ﻿Dubrava	2022	M	62	﻿ Left neck pain, Compression Symptoms	2.5	DWD	?	-
[19] Bashir	2021	M	69	Mass, Cervical LMA	?	?	?	-
[20] Akata	1998	F	65	Painless Swellings	< 1	?	?	-
[21] Lida	1993	F	72	Mass	﻿2	DWD after 51 months, NLM	Surgery	-
[22] Amal	2013	F	72	Mass	5	DWD after 2 months	Surgery	-
[23] Canu	2018	M	47	Mass, Compression Symptoms	6	Alive, Recurrence	Surgery, Chemotherapy	-
[24] Conzo	2014	M	77	Mass, Compression Symptoms	4.5–6.5	DWD after 40 days	Surgery	-
[25] Kawahara	1988	M	82	Mass	5.5	DWD after 4 months, LA	Surgery	-
[26] Ozaki	1997	F	58	Mass	5	Alive	Surgery	-
[27] Chetty	1993	F	54	?	3.5	Alive	Surgery	-
[28] Kaur	2022	F	55	Mass	11.9	DWD after 2 weeks	Surgery	-
[29] Tsugawa	1999	F	90	Mass, Compression Symptoms	?	?	Surgery	-
[30] Day	2007	M	43	Mass	6	Recurrence	Surgery	-
[31] Ege	2013	M	56	Mass, Compression Symptoms	﻿3	DWD	Surgery	-
[32] Gupta	2017	F	65	Mass, Compression Symptoms	﻿8.3	?	Surgery	-
[33] Kushnir	2018	F	67	Compression Symptoms	1.6, 2.5	DWD, Recurrence	Surgery	-
[34] Reddy	2019	F	50	﻿Mass	﻿5.9	Alive, Recurrence	Surgery, Radiotherapy, Chemotherapy	-
[35] ﻿Takayama	2001	F	66	﻿Mass	﻿8.5	Recurrence	Surgery	-
[36] Tanboon	2013	F	64	Mass, Compression symptoms	﻿7	DWD after 3 months, NLM	Surgery	-
[37] Wei	2019	F	74	Mass	﻿7	DWD after 2 months	Surgery	-
[38] Ayadi	2017	F	32	Mass	5	?	Surgery, Radiotherapy, Chemotherapy	-
[39] Bertelli	2010	M	39	Mass, Compression symptoms	3.5	Alive	Surgery, Radiotherapy	-
40 Our Case	2022	F	42	Mass	4.5	Alive	Surgery	Concurrent PTC, Uterine Fibroids

*DWD * Dead with disease,  *NLM * Non-lymph node metastasis,  *LA * Locally Advanced,  *LM * Lymph Node metastasis,  *PED * Pediatric,  *EBV * Epstein Barr Virus,  *LMS * Leiomyosarcoma,  *PTC * Papillary Thyroid Cancer

Most LMS cases present with a neck mass (85% of the cases), followed by compressive symptoms (45% of the cases). Other symptoms include weight loss [5, 13–16], arm pain [10, 17], neck pain [17], and cervical lymphadenopathy [19]. Akata et al. reported a female case who presented with painless swellings in her body, and she was diagnosed with multicentric synchronous leiomyosarcomatosis due to having multiple nodules in seemingly unrelated organs involving thyroid and salivary glands, pancreas, ligamentum teres, and bones [20]. Regarding the outcome, 55% of the cases were dead with the disease, with the longest duration of survival being 51 months after the diagnosis [21]. Only 15% of the cases had a recurrence of LMS. Furthermore, 25% of the cases reported non-lymph node metastasis most commonly involving the lungs, while 10% of the cases had lymph node metastasis. Ramakrishnan et al. reported an interesting pediatric case who presented with a neck mass, and elevated serum calcitonin, raising a suspicion of medullary thyroid carcinoma [7]. However, histopathology showed an absence of amyloid stroma, and LMS was diagnosed based on immunohistochemistry. Most of the patients (75%) underwent surgery, and 12.5% of the cases received radiotherapy.

The differential diagnosis of thyroid LMS ranges from benign conditions like cysts and adenomas to extremely malignant conditions like anaplastic carcinoma and metastatic lesions. LMS remains an extraordinarily rare tumor of the thyroid, with our case presenting a concurrent PTC. Concurrent PTC tumors with other thyroid pathologies are generally considered coincidental. Thus, Concurrent PTC tumors are regarded as clinically insignificant by expert consensus; however, concrete literature analyses have not yet demonstrated a clear answer. This report demonstrates the importance of being vigilant of the potential diagnosis, especially with immunohistochemical staining. Physicians should look for possible sources of metastasis before labeling a tumor as primary, as the management varies quite heavily.

## Data Availability

Our data is presented as a table summary in the manuscript.

## Abbreviations

LMS: Leiomyosarcoma
PTC: Papillary Thyroid Cancer
FNA: Fine Needle Aspiration
TSH: Thyroid Stimulation Hormone
TIRADS: Thyroid Nodule Image Reporting and Data Systems
